# Identification of three Asian otter species (*Aonyx cinereus*, *Lutra sumatrana*, and *Lutrogale perspicillata*) using a novel noninvasive PCR‐RFLP analysis

**DOI:** 10.1002/ece3.9585

**Published:** 2022-12-12

**Authors:** Sandeep Sharma, Woo Chee‐Yoong, Adrian Kannan, Suganiya Rama Rao, Pazil Abdul‐Patah, Shyamala Ratnayeke

**Affiliations:** ^1^ German Centre for Integrative Biodiversity Research (iDiv) Halle‐Jena‐Leipzig Leipzig Germany; ^2^ Institute of Biology, Martin Luther University Halle‐Wittenberg Halle Germany; ^3^ Department of Biological Sciences Sunway University Selangor Darul Ehsan Malaysia; ^4^ Malaysian Nature Society Kuala Lumpur Malaysia; ^5^ Department of Wildlife and National Parks (PERHILITAN), Peninsular Malaysia Kuala Lumpur Malaysia; ^6^ Skidmore College New York Saratoga Springs USA

**Keywords:** *Aonyx cinereus*, *Lutra sumatrana*, *Lutrogale perspicillata*, mitochondrial DNA, noninvasive monitoring

## Abstract

Four species of otters occur in tropical Asia, and all face multiple threats to their survival. Studies of distribution and population trends of these otter species in Asia, where they occur sympatrically, are complicated by their elusive nature and difficulties with reliable identification of species in field surveys. In Malaysia, only three species, the smooth‐coated otter, Asian small‐clawed otter, and hairy‐nosed otter have been reliably reported as residents. We designed a replicable and cost‐efficient PCR‐RFLP protocol to identify these three species. Using published reference sequences of mitochondrial regions, we designed and tested three PCR‐RFLP protocols on DNA extracted from reference samples and 33 spraints of wild otters collected along the North Central Selangor Coast of Malaysia. We amplified and sequenced two fragments (450 and 200 bp) of the mt D‐loop region and a 300‐bp fragment of the mt ND4 gene using primer sets TanaD, TanaD‐Mod, and OTR‐ND4, respectively. Amplification products were digested with restriction enzymes to generate species‐specific RFLP profiles. We analyzed the costs of all three protocols and compared these with the costs of sequencing for species identification. Amplification success was highest for the smallest PCR product, with the TanaD‐Mod primer amplifying DNA from all 33 spraints. TanaD and OTR‐ND4 primers amplified DNA from 60.6% and 63.6% spraints, respectively. PCR products of TanaD‐Mod provided the expected species‐specific RFLP profile for 32 (97%) of the spraints. PCR products of OTR‐ND4 provided the expected RFLP profile for all 21 samples that amplified, but TanaD produced spurious bands and inconsistent RFLP profiles. The OTR‐ND4 primer–enzyme protocol was the least expensive (437 USD) for processing 100 samples, followed by TanaD‐Mod (455 USD). We suggest the use of both OTR‐ND4 and TanaD‐Mod protocols that show potential for highly efficient and reliable species identification from noninvasive genetic sampling of three Asian otter species. We expect our novel noninvasive PCR‐RFLP analysis methods to facilitate population monitoring, ecological and behavioral studies on otters in tropical and subtropical Asia.

## Graphical abstract

A group of the Asian small‐clawed otter (*Aonyx cinereus*) in their tidal habitat of the Sundarban mangrove forest in Bangladesh. ©Sandeep Sharma.

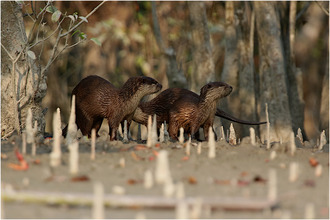

## 1 INTRODUCTION

**FIGURE 1:**
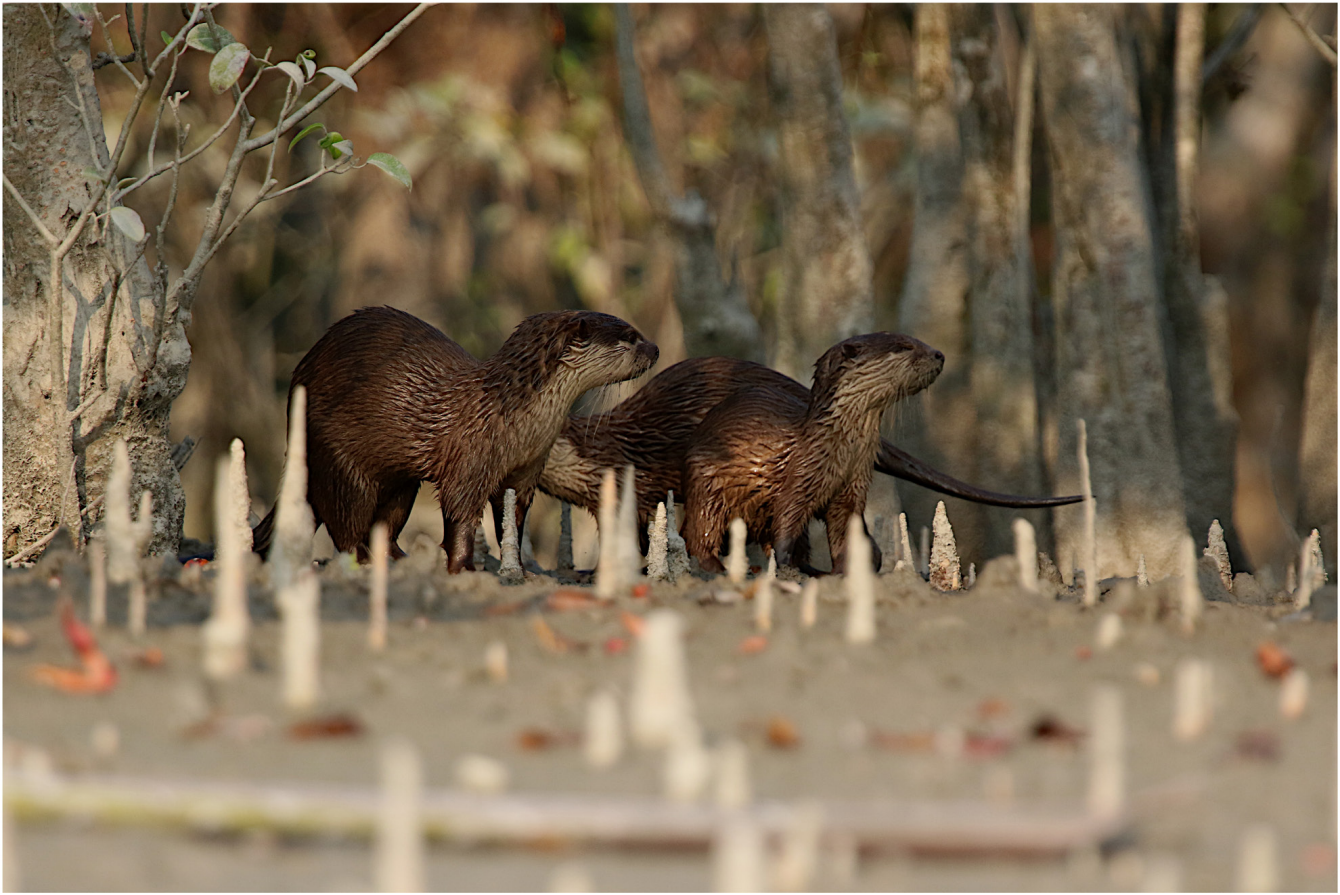
A group of the Asian small‐clawed otter (*Aonyx cinereus*) in their tidal habitat of the Sundarban mangrove forest in Bangladesh.

South‐East Asia, a global biodiversity hotspot, is home to four species of otters: smooth‐coated otter (*Lutrogale perspicillata*), Asian small‐clawed otter (*Aonyx cinereus*; Figure 1), hairy‐nosed otter (*Lutra sumatrana*), and Eurasian otter (*Lutra lutra*; Basnet et al., 2020). Among these four species, the smooth‐coated otter and the Asian small‐clawed otter are listed as Vulnerable, the hairy‐nosed otter as Endangered, and the Eurasian otter as Near‐Threatened in the IUCN's Red List assessment (Khoo et al., 2021; Roos et al., 2021; Sasaki et al., 2021; Wright et al., 2021). There is a dearth of information about the distribution and conservation status of these four species of otters in South‐East Asia (Duplaix & Savage, 2018), where populations are threatened by rapid economic development, habitat loss, illegal wildlife trade, pollution and degradation of aquatic habitat, human‐otter conflict, and climate change (Cianfrani et al., 2018; Duckworth & Hill, 2008; Foster‐Turley, 1992). Increased pollution of aquatic habitats has impacted otter food resources and expanding linear infrastructure such as road networks is emerging as one of the major causes of otter mortality (Peterson & Schulte, 2016).

Increasing threats to otter populations throughout the world have called for increased and focused efforts to monitor their status and distributions (Duplaix & Savage, 2018). However, due to their elusive habits and rarity in distribution, monitoring otters is not an easy task. A plethora of noninvasive survey techniques, which possess the advantages of being relatively inexpensive and replicable, exist to monitor small carnivores (Long et al., 2012). Sign surveys using footprints and spraints have been traditionally used for monitoring presence, population trends, and habitat use, specifically for the Eurasian otter and North American River otter (*Lontra canadensis*). Otter spraints are typically deposited at latrine sites located along the banks of rivers and wetlands. Fresh otter spraints are easily detected by their strong fishy odor. In the last two decades, advancements in camera trapping and noninvasive DNA analysis techniques have opened new avenues for otter monitoring. Camera traps at latrine sites have provided valuable data on species, group size, and social behavior (Ben‐David et al., 1998; Green et al., 2015; Stevens & Serfass, 2008). Noninvasive molecular genetic sampling using spraints has been successfully employed in ecological and demographic studies of Eurasian and North American otters (Hájková et al., 2009; Jansman et al., 2001; Klütsch & Thomas, 2018; Sittenthaler et al., 2020). These methods increase the scope of population monitoring by providing data on otter presence and habitat use, including additional information such as individual identification by DNA analysis, which can be used to estimate population size and density.

Accurate species identification is vital for population monitoring. However, where the ranges of multiple species of otters overlap, as in South‐East Asia, surveys using sign or camera traps are limited by uncertainty and difficulties in definitively assigning spraints, footprints, or photographs to species. Kruuk et al. (1993) and Kistner et al. (2022) provide detailed descriptions to distinguish among tracks, spraints, and spraint sites of three otter species (*Lu. lutra*, *A. cinereus*, and *L. perspicillata*), but these signs, specifically tracks, persist for a short period in tropical weather conditions, and their reliable interpretation depends largely on the consistency of the substrate. A majority of South Asian and South‐East Asian countries have more than two otter species. Cambodia, Indonesia (Sumatra), Thailand, Myanmar, and Vietnam report four species. Thus, efficient and cost‐effective methods to overcome the challenge of species identification are crucial for monitoring otter populations in these regions.

Although all four species of otters have been reported from Malaysia, only three species, the smooth‐coated otter, Asian small‐clawed otter, and hairy‐nosed otter, have consistently confirmed distribution records. Smooth‐coated otters and Asian small‐clawed otters are the most common and occur through much of tropical Asia including Peninsular Malaysia (Abdul‐Patah et al., 2020; Wright et al., 2021) and Malaysian Borneo (Forest Department Sarawak, 2020; Khoo et al., 2021). The hairy‐nosed otter is the only species of otter endemic to South‐East Asia, but is rare with sporadic records from its known range, including Malaysia (Sasaki et al., 2021). A recent study (Abdul‐Patah et al., 2020) provided the most comprehensive information on the spatial distribution, associated habitats, and genetic diversity (mitochondrial) of the three species of otters in Peninsular Malaysia. This study confirmed otter species in 138 locations of Peninsular Malaysia, out of which hairy‐nosed otters were only confirmed from seven sparsely distributed locations. Roadkills of the hairy‐nosed otter have been reported near peat swamp forests in Perak, Pahang (Sebastian, 1995), and more recently in Northern Selangor, Malaysia (Tan, 2015; Woo, 2021). The presence of the Eurasian otter in Malaysia is highly uncertain and limited to just two records from the northern part of the peninsula (Miller, 1900; Mohd‐Azlan & Sharma, 2006) and one from Borneo (Phillipps & Phillipps, 2016). All species of otter are “Totally Protected” based on the Wildlife Conservation Act 2010 in Peninsular Malaysia, and “Protected” in Malaysian Borneo by the Wildlife Conservation Enactment 1997 of the state of Sabah and the Wild Life Protection Ordinance 1998 of the state of Sarawak.

Species identification by DNA analysis of scat samples has relied mainly on the amplification of mitochondrial DNA sequences, such as the control and 16s rRNA regions (e.g., Mills et al., 2000) cytochrome b gene (e.g., Hsieh et al., 2001; Madisha et al., 2015) and mitochondrial D‐loop region (Bozarth et al., 2010). DNA from noninvasive sources such as hair and feces usually contain only trace amounts of low‐quality fragmented DNA, which degrades further with time, resulting in amplification failure in old decayed samples (Shih et al., 2017; Waits & Paetkau, 2005). Abdul‐Patah et al. (2014, 2020) successfully identified otter species from spraints by sequencing a fragment of ~450‐bp DNA, amplified from the mitochondrial D‐loop region. They were successful in amplifying DNA from ~50% of the spraint samples (A.‐P. Pazil, personal communication) to ascertain species. The amplification of short (<300 bp) fragments of mitochondrial DNA is reported to produce higher amplification success rates (Madisha et al., 2015; Shih et al., 2017). PCR amplification of mitochondrial fragments has been successfully combined with restriction‐fragment‐length polymorphisms (PCR‐RFLP) analysis to distinguish among closely related taxa (e.g., Bidlack et al., 2007; Cossíos & Angers, 2006; Hansen & Jacobsen, 1999; Mukherjee et al., 2010). This approach has the advantages of being relatively rapid and cost‐effective because amplification products can be separated by size, producing distinct species‐specific band patterns and eliminating the need for sequencing. Scats deposited by other small carnivores may sometimes be misidentified as otter spraints. Erroneous identification of scats of American mink (*Neovison vison*), European mink (*Mustela lutreola*), and European polecat (*Mustela putorius*) as those of European otter is reported (Hansen & Jacobsen, 1999), but no such information is reported by studies on Asian otter species.

Otters are apex carnivores in aquatic habitats and thus crucial for regulating aquatic food webs and ecosystem‐level trophic dynamics (Ben‐David et al., 1998; Kruuk, 2006). Tracking the status of otter populations is therefore fundamental to ensure the stability of aquatic ecosystems in South and South‐East Asia. Conservation monitoring programs for elusive species such as otters require significant effort to achieve sufficient sample sizes for reliable estimates. Higher amplification success rates and accurate species identification with noninvasive samples will greatly improve the quality of data needed for accurate mapping of otter species range, population distributions, estimation of occupancy, density, and population trends. Here, we develop and evaluate three PCR‐RFLP (polymerase chain reaction—restriction‐fragment‐length polymorphism) protocols with the aim of designing an efficient and cost‐effective noninvasive species identification method for three otter species found in Malaysia and other parts of South‐East Asia. Further, we compare these PCR‐RFLP protocols for their cost‐effectiveness and their ability in successfully and reliably identifying these otter species.

## 2 MATERIALS AND METHODS

### 2.1 Field sampling and reference sample collection

**FIGURE 2:**
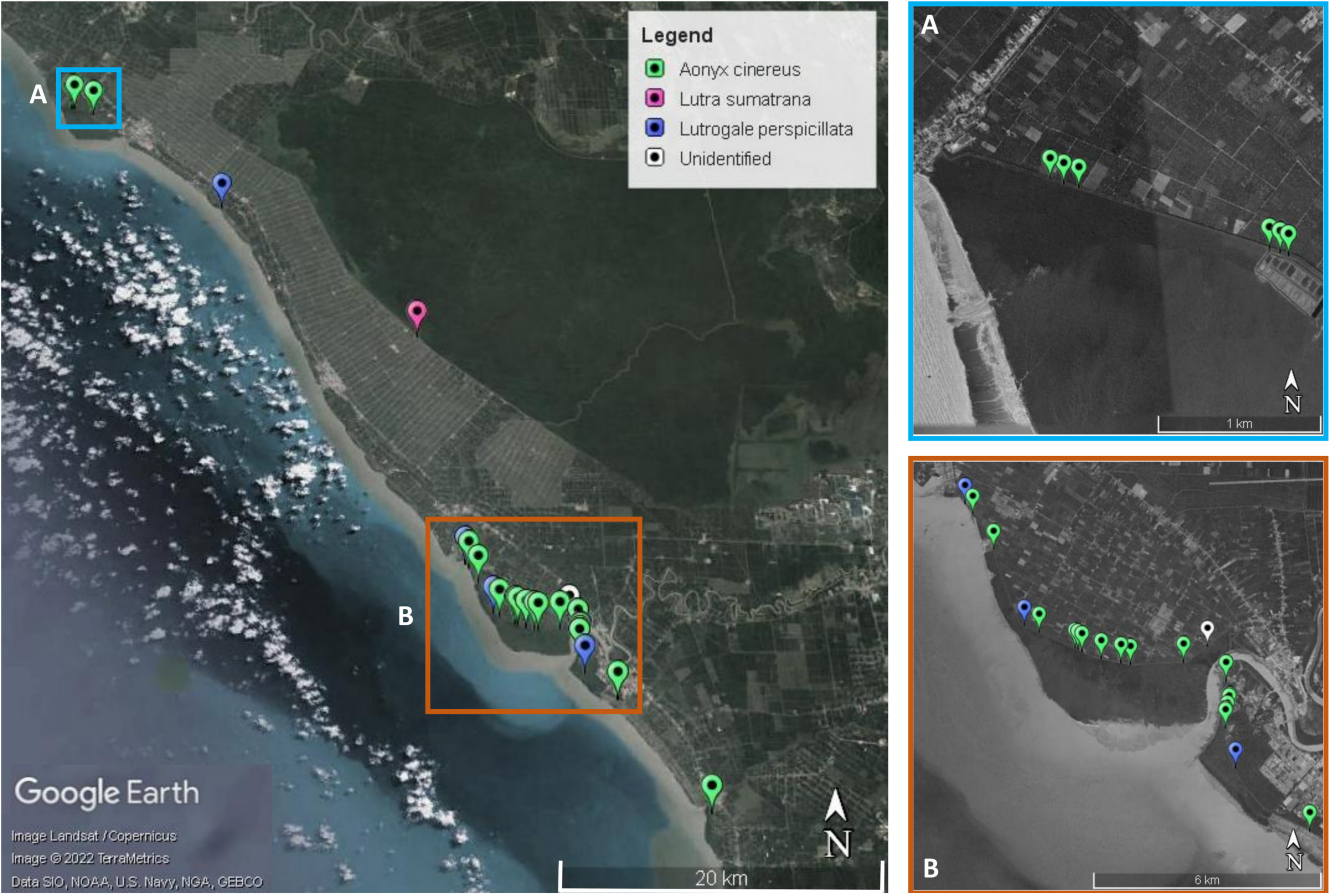
Map of the North Central Selangor Coast in Peninsular Malaysia, where 33 otter spraint samples were collected for our study. Insets A and B are magnified views of sampled sections of North Central Selangor Coast. The identification and assignment of these samples to otter species are based on PCR‐RFLP results of this study.

This study is the preliminary part of a larger project to systematically survey and map the distribution of otters along the North Central Selangor Coast (Lat 3.8444° N, Long 101.8152° E to Lat 2.9762° N, Long 101.2811° E) on the west coast of Malaysia (Figure 2). Opportunistic records and surveys confirm that all three species of otter inhabit this region (Abdul‐Patah et al., 2020; Woo, 2021). We collected 33 otter spraint samples from various locations in this region (Figure 2). We obtained reference samples (blood samples on FTA cards and/or tissue samples) of two species of otters (smooth‐coated otter and Asian small‐clawed otter), common palm civet (*Paradoxurus hermaphroditus*), and short‐tailed mongoose (*Herpestes brachyurus*) from the Department of Wildlife and National Parks, Peninsular Malaysia (PERHILITAN; Table S1). The common palm civet and short‐tailed mongoose are two small carnivores that occur sympatrically with otters in the North Central Selangor Coast. Their scats are similar in size to those of otters, although chances of scat misidentification are generally minimal due to morphological dissimilarities between scats and spraints and dietary differences among these species.

We surveyed potential otter habitat to locate spraint sites and collected fresh spraints in 5‐ml collection vials with absolute ethanol as preservative. These samples were stored at room temperature/−20°C before lab analysis.

### 2.2 Laboratory analysis

We extracted DNA from approximately 0.25 mg of spraint samples via the alkaline lysis method using the GF‐1 Soil Sample DNA Extraction Kit (Vivantis Technologies). DNA extraction procedure followed the manufacturer's instructions with a slight modification; the incubation time was increased to 5 min prior to DNA elution to maximize DNA yield. We also extracted DNA from reference samples of *A. cinereus*, *L. perspicillata*, and *H. brachyurus* from blood samples stored on the FTA cards and *P. hermaphroditus* from tissue samples using the GF‐1 Tissue Blood Combi DNA Extraction Kit (Vivantis Technologies). The extracted DNA was stored at −20°C.

We amplified two fragment sizes of the mt D‐loop region using two different sets of primers: (i) forward TanaD‐F, 5′‐ACCATCAGCACCCAAAGCTG‐3′ (Masuki et al., 2008) and reverse TanaD‐R, 5′‐GGGCTGATTAGTCATTAGTCCATC‐3′ (Masuki et al., 2008) primers for amplification of 450‐bp fragment and (ii) forward TanaD‐Mod‐F (5′‐CACCATGCCTCGAGAAACCA‐3′) designed in this study and reverse TanaD‐R primers for a shorter 200‐bp fragment. Additionally, we designed another set of primers (forward OTR‐ND4‐F, 5′‐ GGCAACCAAACAGAACGCC‐3′ and reverse OTR‐ND4‐R, 5′‐ GTTTAAGGAGTACGGCGGCAA‐3′) to amplify a 300‐bp fragment of the mt ND4 gene.

We designed the OTR‐ND4 primer using Primer‐Blast (Ye et al., 2012) along with the complete mitochondrial genomes of three otter species (*A. cinereus*: GenBank KY117535.1, *L. perspicillata*: GenBank KY117557.1, *Lu. sumatrana*: GenBank KY117556.1; Salleh et al., 2017). We used Primer‐Blast to design the TanaD‐Mod‐F primer using 324 published sequences (Abdul‐Patah et al., 2020) representing all three species (*A. cinereus*: GenBank OL588639.1–OL588743.1, *L. perspicillata*: GenBank OL588744.1–OL588954.1, *Lu. sumatrana*: GenBank OL588632.1–OL588638.1; Appendix 1). We used the OligoAnalyzer™ Tool (Integrated DNA Technologies) to check for secondary structures in these primers. Thereafter, we searched for appropriate sets of restriction enzymes using an interactive web‐based program VIRS (Chen et al., 2009). This tool can identify and visualize restriction sites in multiple DNA sequences and suggests appropriate restriction enzymes for those sites. Our objective was to select those enzymes that would not be affected by CpG methylation and provide discernible band patterns (ideally less than three bands with sufficient difference among their size) for easy visualization on agarose gels. We also conducted in silico PCR and restriction digestion, followed by gel visualization in the SnapGene program (GSL Biotech LLC).

DNA amplifications of the targeted fragments were conducted in a final reaction volume of 25 μl consisting of 12.5 μl ExPrime Taq Premix (2X) (GENETBIO Inc.), 7.5 μl distilled water, 1.5 μl of 10 pmol/μl forward and reverse primers, and 2 μl extracted otter fecal DNA. PCR was carried out using T100® Thermal Cycler (Bio‐Rad Laboratories) with the following parameters: initial denaturation at 94°C for 10 min followed by 45 cycles of denaturation at 94°C for 30 s, annealing at 56°C and 62°C for D‐loop fragments and ND4 fragment, respectively, for 30 s, and extension at 72°C for 1 min, and a final extension at 72°C for 7 min. The amplicons were electrophoresed and visualized on a 2% agarose gel to determine specific amplifications. We included extraction negatives and PCR negatives in every batch of PCR performed.

First, we confirmed all reference samples, including one spraint sample of hairy‐nosed otter collected from our study area that was used as a reference for all follow‐up lab analysis. These samples were sequenced (Applied Biosystems™ 3730xl DNA Analyzer) for the mt D‐loop and mt ND4 regions with the primers mentioned above. Forward and reverse sequences were checked, edited, and merged into contigs using ChromasPro2.0 (Technelysium Pty. Ltd.). Sequences obtained from this study were compared with reference taxonomic sequences deposited in the National Center for Biotechnology Information (NCBI) using the nucleotide Basic Local Alignment Search Tool (nBLAST) with default settings. Species‐specific amplifications were confirmed if query sequences shared high similarity with any taxa from the Lutrinae subfamily. After confirming species identity, these reference samples were used as positive controls for subsequent PCR reactions of 33 spraint samples collected from various sites in the study region.

After confirming the amplification of PCR products, restriction digestions were conducted in a final reaction volume of 30 μl consisting of 10 μl PCR products, 1–2 μl restriction enzyme (Thermo Fisher Scientific), 2 μl 1X buffer (Thermo Fisher Scientific), and 16–17 μl distilled water. The digestion mixtures were incubated at 37°C (*Hin*1II, *Nde*I, and *Bsu*RII) and 65°C (*Mse*I and *Hpy*CH4III) for 4 h, and digested products were separated via electrophoresis on a 2.5% agarose gel at 60 V for 2 h.

### 2.3 Cost analysis

Our aim was to develop a cost‐effective and accurate protocol that would be useful and financially feasible to otter researchers in Asia. Thus, we evaluated the cost of the reagents required for processing a batch of 100 samples to determine the most cost‐effective approach for species identification of three species of otters using the PCR‐RFLP method with three primer sets and a combination of suitable restriction enzymes. We also compared the cost of PCR‐RFLP with sequencing costs for species identification. Reagents used in this study were commercially available and were obtained from local distributors in Malaysia. We estimated and converted the cost in U.S. Dollars (US$).

## 3 RESULTS

**FIGURE 3:**
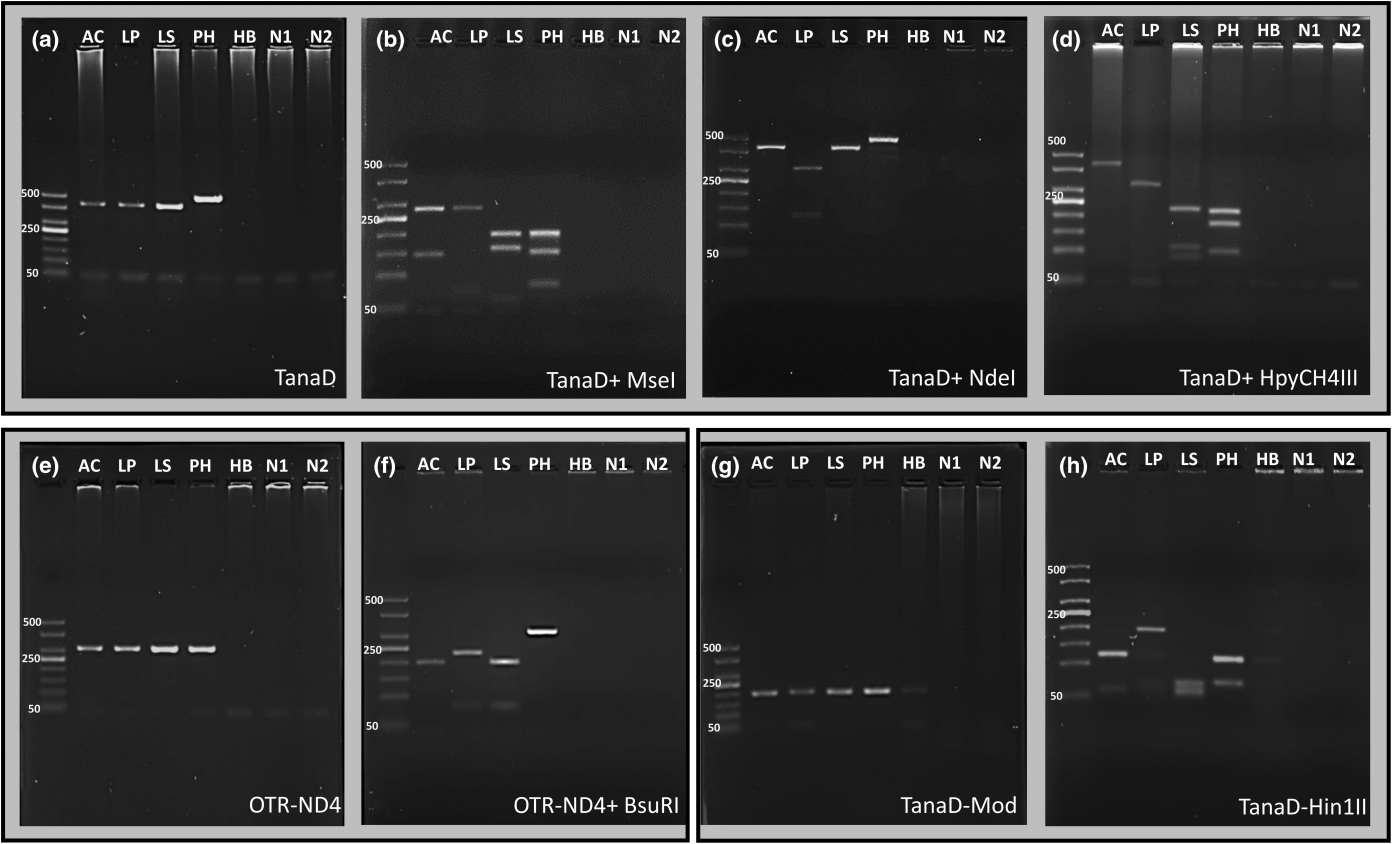
Gel electrophoresis results of successful amplification of *Aonyx cinereus* (AC), *Lutrogale perspicillata* (LP), *Lutra sumatrana* (LS), *Paradoxurus hermaphroditus* (PH), and *Herpestes brachyurus* (HB) reference samples using three primers sets (TanaD, OTR‐ND4, and TanaD‐Mod) and combinations of restriction enzymes for reference samples of *A. cinereus*, *L. perspicillata*, *Lu. sumatrana*, *P. hermaphroditus*, and *H. brachyurus*, respectively. The two negative wells (N1 and N2) refer to DNA extraction and PCR negatives, respectively. Panels (a–h) show results of PCR amplification and RFLP digestions of three primers and restriction enzymes.

Sequencing confirmed that all reference samples were from their respective species. Reference samples for three otter species, including the spraint sample of one hairy‐nosed otter and the common palm civet, amplified successfully with the three primer sets (TanaD, TanaD‐Mod, and OTR‐ND4) used in this study and were ascertained to their respective species by an nBLAST search (Figure 3a,e,g). We also found that the fragment size for the common palm civet reference sample was larger (~500 bp) than those of the otter species (~450 bp; Figure 3a). The short‐tailed mongoose reference sample amplified only with the TanaD‐Mod primer, providing an amplification product similar in size to the other four species (Figure 3g), but did not produce any distinct fragments in RFLP analysis (Figure 3h). RFLP analysis of the remaining four species with three sets of primers and five restriction enzymes provided species‐specific band patterns (Figure 3b–d,f,h).

Amplification rates of 33 samples differed among the three primer sets. Amplification success decreased with increasing PCR fragment size. The TanaD primer with the largest PCR product (~450 bp) amplified just 60.6% (*n* = 20) of DNA from spraints and produced spurious bands of various sizes for several samples. This primer set also produced inconsistent results for RFLP analysis with three different restriction enzymes *Mse*I, *Nde*I, and *Hpy*CH4III. Therefore, we discontinued the use of TanaD to analyze further samples using the PCR‐RFLP method. Amplification rates were slightly better (63.6% of spraints) for the OTR‐ND4 primer, which produced a band of ~300 bp. Moreover, OTR‐ND4 provided the expected RFLP profile for correct species identification after RFLP analysis with *Bsu*RI for all 21 spraint samples that successfully amplified. The TanaD‐Mod primer, which yielded the smallest PCR product (~200 bp) of all three primer sets, amplified all 33 samples. Subsequent RFLP analysis with *Hin*1II restriction enzyme identified the species of otter in 32 (97%) of the samples.

We compared the costs of PCR‐RFLP analysis for the three protocols. The OTR‐ND4 primer and *Bsu*RI enzyme pair was the least expensive, totaling 437 USD for DNA extraction, PCR amplification, and RFLP analysis of 100 samples (Table 1). The TanaD‐Mod primer with *Hin*1II enzyme would cost 455 USD for analysis. However, if compared both for consistency and accuracy in species identification and costs, the TanaD‐Mod and *Hin*1II pair emerges as the most cost‐effective PCR‐RFLP protocol (Table 2).

**TABLE 1:** Detailed cost breakdown of lab analysis.

Lab analysis	TanaD‐Mod	TanaD	TanaD			Sequencing
	*Hin1*II	*Bsu*RI	*Mse*I	*Nde*I	*Hpy*CH4III	
DNA extraction	349.4	349.4	349.4	349.4	349.4	349.4
Primer	14.7	13.4	14.7	14.7	14.7	14.7
PCR Premix	45.3	45.3	45.3	45.3	45.3	45.3
Other material^*^	26.8	26.8	26.8	26.8	26.8	26.8
Restriction Enzyme	17.9	2.0	16.5	5.0	33.4	0.0
Sequencing						501.2
Total cost	455	437	453	442	470	937

*Note*: Costs were calculated for the analysis of 100 samples and reported in the USD. Various colors (orange, green and blue) indicate different stages of laboratory analysis.

* Agarose, TBE buffer, DNA stain, loading dye, and plastic consumables.

**TABLE 2:** Comparison of amplification success, PCR‐RFLP accuracy in species identification, and cost comparison of various primer sets and Sanger sequencing used in our study.

Primers	TanaD‐Mod	OTR‐ND4	TanaD			Sequencing
Amplification success (%)	100	63.63	60.6			
**Restriction enzymes**	***Hin*1II**	***Bsu*RI**	***Mse*I**	***Nde*I**	***Hpy*CH4II**	
PCR‐RFLP accuracy (%)	96.97	61.9	30	–	–	
Cost, 100× (US$)	455	437	453	442	470	937

*Note*: Cost per 100 samples includes chemical consumables for DNA extraction, PCR amplification, and RFLP analysis.

## 4 DISCUSSION

Monitoring small carnivores is a daunting task because of their elusive nature and sparse distribution. Where closely related species of small carnivores co‐occur, signs such as tracks and scats are often not very useful to ascertain species. Remote cameras have improved the quality of small carnivore surveys, but have limitations where morphologically similar species such as Asian otters co‐occur. There is an enormous lack of information about small carnivore distribution and ecology, with no robust and scientifically validated information on distribution ranges and population size for a number of species, owing to the lack of research studies (Glatston & Duplaix, 2020; Torres‐Romero & Giordano, 2022). Without this baseline information, assessment of the impacts and extent of threats to these species and the effectiveness of conservation measures is difficult.

The primary goal of this study was to develop an accurate, easy‐to‐implement, cost‐effective DNA analysis method for the identification of three Asian otter species. We describe a cost‐effective PCR‐RFLP method for identifying three otter species that has very high amplification success with noninvasive samples and is half the cost of Sanger sequencing for species identification. We also detected an inverse relationship between fragment size and amplification success rates for the three primers in this study, which has also been reported in previous studies (Broquet et al., 2007; Deguilloux et al., 2002, 2003; Shih et al., 2017). Previously, the TanaD primer was used for amplifying DNA from otter spraints followed by Sanger sequencing to identify species (Abdul‐Patah et al., 2020). Besides the high costs and technical expertise required for sequencing analysis, amplification success was low (~50%) in the study due to the large fragment size. In our study, RFLP analysis of the amplified PCR product of TanaD primer produced inconsistent results; thus, we consider it unsuitable for species identification using PCR‐RFLP on noninvasive samples. Amplification success of large fragments is expected to be low with noninvasive samples such as spraints in which DNA is likely to degrade rapidly in warm, humid tropical environments. We designed a new forward primer, TanaD‐Mod‐F, to amplify a shorter fragment of the TanaD primer with the expectation that the smaller fragment length of the PCR product would increase amplification success of spraint samples. As expected, this modified primer yielded a very high amplification rate (99.3%) with field‐collected samples.

One of the strengths of our study is that we used a published database of 324 reference sequences for three otter species (*Lu. sumatrana*, *A. cinereus*, and *L. perspicillata*) to select suitable restriction enzymes that had unique restriction sites for all the haplotypes reported for these three species in Malaysia (Abdul‐Patah et al., 2020). This approach increased the likelihood that the selected primer–enzyme pair would produce an accurate RFLP profile while also ensuring replicability by checking for the possibility of mutations at enzyme cleavage sites by screening these 313 reference sequences collected from various locations in Malaysia. The D‐loop region of mitochondrial DNA is highly variable, and any PCR‐RFLP assay designed for this region with a few reference sequences might have a risk of nonreplicability when applied to samples collected from other regions. To further address this issue, we tested the TanaD‐Mod primer with 151 field‐collected spraint samples and amplified 99.3% of these samples (Sunway University, unpublished data). Of these, 84.1% (*n* = 127) were identified as spraints deposited by otter species and 15.9% (*n* = 24) as those of common palm civet. We designed the OTR‐ND4 primer for a second primer–enzyme combination targeting the mitochondrial ND4 region of the three otter species. Although this primer–enzyme pair had less amplification success than TanaD‐Mod, it had moderately high accuracy and was the least expensive among all three PCR‐RFLP protocols. We suggest the use of the OTR‐ND4 primer in conjunction with the TanaD‐Mod primer–enzyme pair as a complimentary marker where species identification needs to be reconfirmed (e.g., forensic analysis of confiscated samples).

It is prudent to mention here that hybridization between *L. perspicillata* and *A. cinereus* has been reported in the putative otter sample of *L. perspicillata* collected from Singapore (Moretti et al., 2017), but this study also mentions that hybridization events such as these are likely to occur when the population of one species is much smaller than the other (*A. cinereus* in this case). This calls for further investigation with nuclear markers to ascertain species identification, particularly if one of the co‐occurring species is rare in the study region.

Cost is an important determinant of the feasibility of any monitoring program. Most genetic monitoring programs require technical knowledge, access to highly expensive lab equipment, and analyses whose costs are often prohibitive for most researchers in South and South‐East Asia. These are important limiting factors for research and conservation work in this region, where local students and wildlife researchers work on shoestring budgets for their projects. The protocols we present in this study were developed specifically to reduce the costs and processing time of molecular genetic species identification. For otter researchers planning large‐scale monitoring programs for otters in Asia, we anticipate that this protocol will improve efficiency and enhance data quality.

We caution that our PCR‐RFLP approach is based on a reference database consisting largely of otter species from the Malay peninsula. In other regions, different RFLP profiles may result because of regional haplotype differences. Thus, we suggest for an initial pilot study to confirm species identity via sequencing as an essential prelude to surveys using the PCR‐RFLP methods described here. Also, our PCR‐FLP approach can be applied through the Malay peninsula and Borneo where Eurasian otter is not a resident. In areas where the Eurasian otter is sympatric with smooth‐coated otters or small‐clawed otters (i.e., ~11 countries in tropical Asia; Khoo et al., 2021; Roos et al., 2021; Wright et al., 2021), we recommend screening spraint samples using Hansen and Jacobsen's (1999) PCR‐RFLP protocol for Eurasian otter species identification for further confirmation of their presence. We also recommend to develop and test other upcoming advanced technique such as e‐DNA analysis with next‐generation sequencing (NGS) for distribution surveys of Asian otter species. New advanced techniques such as NGS hold much promise for noninvasive genetic studies, but may not be available nor affordable for ecological monitoring in several otter range countries in South and South‐East Asia in the near future. The methodologies presented here provide a more affordable alternative.

## 5 CONCLUSION

Otters are sentinels of freshwater ecosystems. They are also resilient species that respond well to habitat conservation and population protection. In places where habitats (rivers, wetlands, and marine coastlines) are restored, and where pollution is controlled, otters have shown remarkable recolonization potential (Duplaix & Savage, 2018). Many of the world's species of otter coexist with people, including in highly urbanized areas such as Singapore (Khoo & Lee, 2020; Theng & Sivasothi, 2016). Making an active effort for otter conservation in these regions is also linked with some of the specific Sustainable Development Goals (SDGs) such as SDG 6—clean water and sanitation, SDG 14—life below water, SDG 15—life on land, and SDG 13—climate action. Asian otters are in peril due to loss of habitat, trafficking for pelts and the pet trade, and from conflicts with aquaculture industries. Conservation measures for these species would require robust, replicable, and cost‐effective monitoring methods. The PCR‐RFLP methods described in this paper match these criteria and can accurately identify three species of Asian otters. These protocols also have important forensic applications such as the identification of seized otter pelts and roadkills. Accurate species identification is also an important step that should precede genetic identification of individuals, sex, relatedness, population genetics, and diet assessment by metabarcoding. The methods we describe here can be used to generate baseline spatial distribution maps, including studies of occupancy, species interaction and habitat use, genetic monitoring, and other valuable ecological information that will facilitate the conservation of otters in Asia.

## AUTHOR CONTRIBUTIONS

**Sandeep Sharma:** Conceptualization (equal); data curation (equal); formal analysis (equal); investigation (equal); methodology (equal); project administration (equal); supervision (equal); validation (equal); visualization (equal); writing – original draft (equal); writing – review and editing (equal). **Woo Chee‐Yoong:** Conceptualization (equal); data curation (equal); funding acquisition (equal); investigation (equal); methodology (equal); project administration (equal); writing – original draft (equal); writing – review and editing (equal). **Adrian Kannan:** Data curation (equal); formal analysis (equal); investigation (equal); methodology (equal); validation (equal); visualization (equal); writing – original draft (equal). **Suganiya Rama Rao:** Data curation (equal); formal analysis (equal); investigation (equal); methodology (equal); validation (equal); visualization (equal). **Pazil Abdul‐Patah:** Resources (equal); supervision (equal); validation (equal). **Shyamala Ratnayeke:** Conceptualization (equal); funding acquisition (equal); investigation (equal); methodology (equal); project administration (equal); supervision (equal); validation (equal); visualization (equal); writing – original draft (equal); writing – review and editing (equal).

## Supporting information


Table S1.

## Data Availability

The data that support the findings of this study are available from the corresponding author upon reasonable request.

## APPENDIX 1 Reference sequence

**Table:**

S. No.	GenBank accession number	Description
1	OL588743.1	*Aonyx cinereus* isolate SG_NIPAH_AC2 D‐loop, partial sequence; mitochondrial
2	OL588742.1	*Aonyx cinereus* isolate PSR_PANJANG_AC_2 D‐loop, partial sequence; mitochondrial
3	OL588741.1	*Aonyx cinereus* isolate PSR_PANJANG_AC_1 D‐loop, partial sequence; mitochondrial
4	OL588740.1	*Aonyx cinereus* isolate SG_Panjang_AC_1 D‐loop, partial sequence; mitochondrial
5	OL588739.1	*Aonyx cinereus* isolate Bgn_Lalang_AC_1 D‐loop, partial sequence; mitochondrial
6	OL588738.1	*Aonyx cinereus* isolate Sg_Pelek_AC_2 D‐loop, partial sequence; mitochondrial
7	OL588737.1	*Aonyx cinereus* isolate Sg_Pelek_AC_1 D‐loop, partial sequence; mitochondrial
8	OL588736.1	*Aonyx cinereus* isolate Ulu_Yam_AC_1 D‐loop, partial sequence; mitochondrial
9	OL588735.1	*Aonyx cinereus* isolate Sg_Buluh_AC_1 D‐loop, partial sequence; mitochondrial
10	OL588734.1	*Aonyx cinereus* isolate Sg_Timun_AC_3 D‐loop, partial sequence; mitochondrial
11	OL588733.1	*Aonyx cinereus* isolate Sg_Timun_AC_2 D‐loop, partial sequence; mitochondrial
12	OL588732.1	*Aonyx cinereus* isolate Sg_Timun_AC_1 D‐loop, partial sequence; mitochondrial
13	OL588731.1	*Aonyx cinereus* isolate LUKUT_AC_1 D‐loop, partial sequence; mitochondrial
14	OL588730.1	*Aonyx cinereus* isolate KPilah_AC_2 D‐loop, partial sequence; mitochondrial
15	OL588729.1	*Aonyx cinereus* isolate KPilah_AC_1 D‐loop, partial sequence; mitochondrial
16	OL588728.1	*Aonyx cinereus* isolate SERTING_AC_3 D‐loop, partial sequence; mitochondrial
17	OL588727.1	*Aonyx cinereus* isolate SERTING_AC_2 D‐loop, partial sequence; mitochondrial
18	OL588726.1	*Aonyx cinereus* isolate SERTING_AC_1 D‐loop, partial sequence; mitochondrial
19	OL588725.1	*Aonyx cinereus* isolate Klinggi_AC_3 D‐loop, partial sequence; mitochondrial
20	OL588724.1	*Aonyx cinereus* isolate Klinggi_AC_2 D‐loop, partial sequence; mitochondrial
21	OL588723.1	*Aonyx cinereus* isolate Klinggi_AC_1 D‐loop, partial sequence; mitochondrial
22	OL588722.1	*Aonyx cinereus* isolate Durian_Tunggal_AC3 D‐loop, partial sequence; mitochondrial
23	OL588721.1	*Aonyx cinereus* isolate Durian_Tunggal_AC2 D‐loop, partial sequence; mitochondrial
24	OL588720.1	*Aonyx cinereus* isolate Durian_Tunggal_AC1 D‐loop, partial sequence; mitochondrial
25	OL588719.1	*Aonyx cinereus* isolate Perlok_AC_3(2) D‐loop, partial sequence; mitochondrial
26	OL588718.1	*Aonyx cinereus* isolate Perlok_AC_3 D‐loop, partial sequence; mitochondrial
27	OL588717.1	*Aonyx cinereus* isolate Perlok_AC_2 D‐loop, partial sequence; mitochondrial
28	OL588716.1	*Aonyx cinereus* isolate Perlok_AC_1 D‐loop, partial sequence; mitochondrial
29	OL588715.1	*Aonyx cinereus* isolate Tmn_Negara_AC_3 D‐loop, partial sequence; mitochondrial
30	OL588714.1	*Aonyx cinereus* isolate K_Rompin_AC_2 D‐loop, partial sequence; mitochondrial
31	OL588713.1	*Aonyx cinereus* isolate Ksalang_AC_1 D‐loop, partial sequence; mitochondrial
32	OL588712.1	*Aonyx cinereus* isolate KSalang_AC_3 D‐loop, partial sequence; mitochondrial
33	OL588711.1	*Aonyx cinereus* isolate KPerlis3_AC_3 D‐loop, partial sequence; mitochondrial
34	OL588710.1	*Aonyx cinereus* isolate KPerlis3_AC_2 D‐loop, partial sequence; mitochondrial
35	OL588709.1	*Aonyx cinereus* isolate KPerlis3_AC_1 D‐loop, partial sequence; mitochondrial
36	OL588708.1	*Aonyx cinereus* isolate KPerlis2_AC_2 D‐loop, partial sequence; mitochondrial
37	OL588707.1	*Aonyx cinereus* isolate KPerlis1_AC_2 D‐loop, partial sequence; mitochondrial
38	OL588706.1	*Aonyx cinereus* isolate KPerlis1_AC_1 D‐loop, partial sequence; mitochondrial
39	OL588705.1	*Aonyx cinereus* isolate Kuala_Muda_AC_4 D‐loop, partial sequence; mitochondrial
40	OL588704.1	*Aonyx cinereus* isolate Kuala_Muda_AC_2 D‐loop, partial sequence; mitochondrial
41	OL588703.1	*Aonyx cinereus* isolate Kuala_muda_AC_1 D‐loop, partial sequence; mitochondrial
42	OL588702.1	*Aonyx cinereus* isolate Kg_hj_jamal_AC_2 D‐loop, partial sequence; mitochondrial
43	OL588701.1	*Aonyx cinereus* isolate Kubang_Ps_AC_2 D‐loop, partial sequence; mitochondrial
44	OL588700.1	*Aonyx cinereus* isolate Kubang_Ps_AC_1 D‐loop, partial sequence; mitochondrial
45	OL588699.1	*Aonyx cinereus* isolate Penaga_AC_1 D‐loop, partial sequence; mitochondrial
46	OL588698.1	*Aonyx cinereus* isolate Penaga_AC_2 D‐loop, partial sequence; mitochondrial
47	OL588697.1	*Aonyx cinereus* isolate P_Remis2_AC_3 D‐loop, partial sequence; mitochondrial
48	OL588696.1	*Aonyx cinereus* isolate P_Remis2_AC_2 D‐loop, partial sequence; mitochondrial
49	OL588695.1	*Aonyx cinereus* isolate P_Remis2_AC_1 D‐loop, partial sequence; mitochondrial
50	OL588694.1	*Aonyx cinereus* isolate P_Remis_AC_1 D‐loop, partial sequence; mitochondrial
51	OL588693.1	*Aonyx cinereus* isolate KGULA_AC_7 D‐loop, partial sequence; mitochondrial
52	OL588692.1	*Aonyx cinereus* isolate ENDAU_AC_2 D‐loop, partial sequence; mitochondrial
53	OL588691.1	*Aonyx cinereus* isolate SEDILI_AC_2 D‐loop, partial sequence; mitochondrial
54	OL588690.1	*Aonyx cinereus* isolate SEDILI_AC_3 D‐loop, partial sequence; mitochondrial
55	OL588689.1	*Aonyx cinereus* isolate RENGIT_AC_1 D‐loop, partial sequence; mitochondrial
56	OL588688.1	*Aonyx cinereus* isolate TG_PIAI_AC_4 D‐loop, partial sequence; mitochondrial
57	OL588687.1	*Aonyx cinereus* isolate TG_PIAI_AC_1 D‐loop, partial sequence; mitochondrial
58	OL588686.1	*Aonyx cinereus* isolate TG_PIAI_AC_9 D‐loop, partial sequence; mitochondrial
59	OL588685.1	*Aonyx cinereus* isolate TG_PIAI_AC_11 D‐loop, partial sequence; mitochondrial
60	OL588684.1	*Aonyx cinereus* isolate TG_PIAI_AC_3 D‐loop, partial sequence; mitochondrial
61	OL588683.1	*Aonyx cinereus* isolate TG_PIAI_AC_2 D‐loop, partial sequence; mitochondrial
62	OL588682.1	*Aonyx cinereus* isolate TG_PIAI_AC_10 D‐loop, partial sequence; mitochondrial
63	OL588681.1	*Aonyx cinereus* isolate PONTIAN_AC_5 D‐loop, partial sequence; mitochondrial
64	OL588680.1	*Aonyx cinereus* isolate PONTIAN_AC_3 D‐loop, partial sequence; mitochondrial
65	OL588679.1	*Aonyx cinereus* isolate PONTIAN_AC_2 D‐loop, partial sequence; mitochondrial
66	OL588678.1	*Aonyx cinereus* isolate G_PATAH_AC_4 D‐loop, partial sequence; mitochondrial
67	OL588677.1	*Aonyx cinereus* isolate G_PATAH_AC_2 D‐loop, partial sequence; mitochondrial
68	OL588676.1	*Aonyx cinereus* isolate G_PATAH_AC_1 D‐loop, partial sequence; mitochondrial
69	OL588675.1	*Aonyx cinereus* isolate SG_BUAYA_AC2 D‐loop, partial sequence; mitochondrial
70	OL588674.1	*Aonyx cinereus* isolate SG_BUAYA_AC1 D‐loop, partial sequence; mitochondrial
71	OL588673.1	*Aonyx cinereus* isolate T_AGAS_AC_8 D‐loop, partial sequence; mitochondrial
72	OL588672.1	*Aonyx cinereus* isolate T_AGAS_AC_4 D‐loop, partial sequence; mitochondrial
73	OL588671.1	*Aonyx cinereus* isolate T_AGAS_AC_3 D‐loop, partial sequence; mitochondrial
74	OL588670.1	*Aonyx cinereus* isolate P_Karang_AC_6 D‐loop, partial sequence; mitochondrial
75	OL588669.1	*Aonyx cinereus* isolate P_Karang_AC_5 D‐loop, partial sequence; mitochondrial
76	OL588668.1	*Aonyx cinereus* isolate Pekan_Nanas_AC_1 D‐loop, partial sequence; mitochondrial
77	OL588667.1	*Aonyx cinereus* isolate Sg_Kemaman_AC_4 D‐loop, partial sequence; mitochondrial
78	OL588666.1	*Aonyx cinereus* isolate Sg_Kemaman_AC_2 D‐loop, partial sequence; mitochondrial
79	OL588665.1	*Aonyx cinereus* isolate Sg_Kemaman_AC_1 D‐loop, partial sequence; mitochondrial
80	OL588664.1	*Aonyx cinereus* isolate Penarik_AC_14 D‐loop, partial sequence; mitochondrial
81	OL588663.1	*Aonyx cinereus* isolate Penarik_AC_13 D‐loop, partial sequence; mitochondrial
82	OL588662.1	*Aonyx cinereus* isolate Penarik_AC_5 D‐loop, partial sequence; mitochondrial
83	OL588661.1	*Aonyx cinereus* isolate Penarik_AC_3 D‐loop, partial sequence; mitochondrial
84	OL588660.1	*Aonyx cinereus* isolate Penarik_AC_2 D‐loop, partial sequence; mitochondrial
85	OL588659.1	*Aonyx cinereus* isolate Sabak_AC_3 D‐loop, partial sequence; mitochondrial
86	OL588658.1	*Aonyx cinereus* isolate Sabak_AC_2 D‐loop, partial sequence; mitochondrial
87	OL588657.1	*Aonyx cinereus* isolate Sabak_AC_1 D‐loop, partial sequence; mitochondrial
88	OL588656.1	*Aonyx cinereus* isolate Kg_Padang_Slm_AC_14 D‐loop, partial sequence; mitochondrial
89	OL588655.1	*Aonyx cinereus* isolate Padang_salimAC_13 D‐loop, partial sequence; mitochondrial
90	OL588654.1	*Aonyx cinereus* isolate AC_Kg_Padang_Salim_12 D‐loop, partial sequence; mitochondrial
91	OL588653.1	*Aonyx cinereus* isolate KgPadang_Salim_AC_11 D‐loop, partial sequence; mitochondrial
92	OL588652.1	*Aonyx cinereus* isolate KgPadang_Salim_AC_10 D‐loop, partial sequence; mitochondrial
93	OL588651.1	*Aonyx cinereus* isolate Kg_Padang_Salim_AC_9 D‐loop, partial sequence; mitochondrial
94	OL588650.1	*Aonyx cinereus* isolate Kg_Padang_Salim_8 D‐loop, partial sequence; mitochondrial
95	OL588649.1	*Aonyx cinereus* isolate KgPadang_Salim_AC_37570_7 D‐loop, partial sequence; mitochondrial
96	OL588648.1	*Aonyx cinereus* isolate KgPadang_Salim_AC_37570_4 D‐loop, partial sequence; mitochondrial
97	OL588647.1	*Aonyx cinereus* isolate Kg_Padang_Salim_AC_3 D‐loop, partial sequence; mitochondrial
98	OL588646.1	*Aonyx cinereus* isolate Kg_Padang_Salim_AC_2 D‐loop, partial sequence; mitochondrial
99	OL588645.1	*Aonyx cinereus* isolate Kg_Padang_Salim_AC_1 D‐loop, partial sequence; mitochondrial
100	OL588644.1	*Aonyx cinereus* isolate Tumpat_AC_15 D‐loop, partial sequence; mitochondrial
101	OL588643.1	*Aonyx cinereus* isolate Tumpat_AC_14 D‐loop, partial sequence; mitochondrial
102	OL588642.1	*Aonyx cinereus* isolate Tumpat_AC_12 D‐loop, partial sequence; mitochondrial
103	OL588641.1	*Aonyx cinereus* isolate Tumpat_AC_303 D‐loop, partial sequence; mitochondrial
104	OL588640.1	*Aonyx cinereus* isolate Tumpat_AC_304 D‐loop, partial sequence; mitochondrial
105	OL588639.1	*Aonyx cinereus* isolate AC_Tumpat_505 D‐loop, partial sequence; mitochondrial
106	OL588954.1	*Lutrogale perspicillata* isolate KgPadang_salim_LP_5 D‐loop, partial sequence; mitochondrial
107	OL588953.1	*Lutrogale perspicillata* isolate KgPadang_salim_LP_6 D‐loop, partial sequence; mitochondrial
108	OL588952.1	*Lutrogale perspicillata* isolate Tg_Piandang2_LP_14 D‐loop, partial sequence; mitochondrial
109	OL588951.1	*Lutrogale perspicillata* isolate Tg_Piandang2_LP_12 D‐loop, partial sequence; mitochondrial
110	OL588950.1	*Lutrogale perspicillata* isolate Tg_Piandang2_LP_13 D‐loop, partial sequence; mitochondrial
111	OL588949.1	*Lutrogale perspicillata* isolate Sg_Besut_LP_6 D‐loop, partial sequence; mitochondrial
112	OL588948.1	*Lutrogale perspicillata* isolate T_Alam_LP_1 D‐loop, partial sequence; mitochondrial
113	OL588947.1	*Lutrogale perspicillata* isolate Paya_Indah1_LP_4 D‐loop, partial sequence; mitochondrial
114	OL588946.1	*Lutrogale perspicillata* isolate Paya_Indah1_LP_3 D‐loop, partial sequence; mitochondrial
115	OL588945.1	*Lutrogale perspicillata* isolate Bkt_Tagar_LP_2 D‐loop, partial sequence; mitochondrial
116	OL588944.1	*Lutrogale perspicillata* isolate Bkt_Tagar_LP_1 D‐loop, partial sequence; mitochondrial
117	OL588943.1	*Lutrogale perspicillata* isolate JUGRA_LP_2 D‐loop, partial sequence; mitochondrial
118	OL588942.1	*Lutrogale perspicillata* isolate JUGRA_LP_1 D‐loop, partial sequence; mitochondrial
119	OL588941.1	*Lutrogale perspicillata* isolate Panchang_Bedana3_LP_17 D‐loop, partial sequence; mitochondrial
120	OL588940.1	*Lutrogale perspicillata* isolate Kelanang_LP‐1 D‐loop, partial sequence; mitochondrial
121	OL588939.1	*Lutrogale perspicillata* isolate SG_MAS_LP‐1 D‐loop, partial sequence; mitochondrial
122	OL588938.1	*Lutrogale perspicillata* isolate Paya_Indah2_LP_10 D‐loop, partial sequence; mitochondrial
123	OL588937.1	*Lutrogale perspicillata* isolate Panchang_Bedana3_LP_13 D‐loop, partial sequence; mitochondrial
124	OL588936.1	*Lutrogale perspicillata* isolate Paya_Indah1_LP_6 D‐loop, partial sequence; mitochondrial
125	OL588935.1	*Lutrogale perspicillata* isolate Paya_Indah1_LP_7 D‐loop, partial sequence; mitochondrial
126	OL588934.1	*Lutrogale perspicillata* isolate Panchang_Bedana3_LP_12 D‐loop, partial sequence; mitochondrial
127	OL588933.1	*Lutrogale perspicillata* isolate Paya_Indah1_LP_5 D‐loop, partial sequence; mitochondrial
128	OL588932.1	*Lutrogale perspicillata* isolate Paya_Indah1_LP_1 D‐loop, partial sequence; mitochondrial
129	OL588931.1	*Lutrogale perspicillata* isolate Paya_Indah1_LP_2 D‐loop, partial sequence; mitochondrial
130	OL588930.1	*Lutrogale perspicillata* isolate Sg_Paka_LP_1 D‐loop, partial sequence; mitochondrial
131	OL588929.1	*Lutrogale perspicillata* isolate Panchang_Bedana3_LP_11 D‐loop, partial sequence; mitochondrial
132	OL588928.1	*Lutrogale perspicillata* isolate Panchang_Bedana3_LP_19 D‐loop, partial sequence; mitochondrial
133	OL588927.1	*Lutrogale perspicillata* isolate Panchang_Bedana3_LP_18 D‐loop, partial sequence; mitochondrial
134	OL588926.1	*Lutrogale perspicillata* isolate Panchang_Bedana3_LP_15 D‐loop, partial sequence; mitochondrial
135	OL588925.1	*Lutrogale perspicillata* isolate Panchang_Bedana1_LP_5 D‐loop, partial sequence; mitochondrial
136	OL588924.1	*Lutrogale perspicillata* isolate Panchang_Bedana3_LP_14 D‐loop, partial sequence; mitochondrial
137	OL588923.1	*Lutrogale perspicillata* isolate P_JAWA_LP_2 D‐loop, partial sequence; mitochondrial
138	OL588922.1	*Lutrogale perspicillata* isolate Panchang_Bedana3_LP_10 D‐loop, partial sequence; mitochondrial
139	OL588921.1	*Lutrogale perspicillata* isolate Panchang_Bedana1_LP_7 D‐loop, partial sequence; mitochondrial
140	OL588920.1	*Lutrogale perspicillata* isolate Sg_Perepet_LP_4 D‐loop, partial sequence; mitochondrial
141	OL588919.1	*Lutrogale perspicillata* isolate Panchang_Bedana1_LP_1 D‐loop, partial sequence; mitochondrial
142	OL588918.1	*Lutrogale perspicillata* isolate Panchang_Bedana1_LP_3 D‐loop, partial sequence; mitochondrial
143	OL588917.1	*Lutrogale perspicillata* isolate Sg_Bewah_LP_3 D‐loop, partial sequence; mitochondrial
144	OL588916.1	*Lutrogale perspicillata* isolate Panchang_Bedana3_LP_7 D‐loop, partial sequence; mitochondrial
145	OL588915.1	*Lutrogale perspicillata* isolate K_Kemaman_LP_1 D‐loop, partial sequence; mitochondrial
146	OL588914.1	*Lutrogale perspicillata* isolate Panchang_Bedana2_LP_4 D‐loop, partial sequence; mitochondrial
147	OL588913.1	*Lutrogale perspicillata* isolate Panchang_Bedana1_LP_8 D‐loop, partial sequence; mitochondrial
148	OL588912.1	*Lutrogale perspicillata* isolate Sg_Bewah_LP_4 D‐loop, partial sequence; mitochondrial
149	OL588911.1	*Lutrogale perspicillata* isolate Panchang_Bedana1_LP_4 D‐loop, partial sequence; mitochondrial
150	OL588910.1	*Lutrogale perspicillata* isolate P_JAWA_LP_3 D‐loop, partial sequence; mitochondrial
151	OL588909.1	*Lutrogale perspicillata* isolate Sg_Perepet_LP_3 D‐loop, partial sequence; mitochondrial
152	OL588908.1	*Lutrogale perspicillata* isolate P_JAWA_LP_4 D‐loop, partial sequence; mitochondrial
153	OL588907.1	*Lutrogale perspicillata* isolate Sg_Bewah_LP_2 D‐loop, partial sequence; mitochondrial
154	OL588906.1	*Lutrogale perspicillata* isolate Sg_Bewah_LP_1 D‐loop, partial sequence; mitochondrial
155	OL588905.1	*Lutrogale perspicillata* isolate K_Kemaman_LP_3 D‐loop, partial sequence; mitochondrial
156	OL588904.1	*Lutrogale perspicillata* isolate Sg_Perepet_LP_1 D‐loop, partial sequence; mitochondrial
157	OL588903.1	*Lutrogale perspicillata* isolate Sg_Besut_LP_5 D‐loop, partial sequence; mitochondrial
158	OL588902.1	*Lutrogale perspicillata* isolate K_Kemaman_LP_4 D‐loop, partial sequence; mitochondrial
159	OL588901.1	*Lutrogale perspicillata* isolate Sg_Cacing_LP_2 D‐loop, partial sequence; mitochondrial
160	OL588900.1	*Lutrogale perspicillata* isolate K_Kemaman_LP_2 D‐loop, partial sequence; mitochondrial
161	OL588899.1	*Lutrogale perspicillata* isolate Sg_Paka_LP_4 D‐loop, partial sequence; mitochondrial
162	OL588898.1	*Lutrogale perspicillata* isolate Sg_Paka_LP_3 D‐loop, partial sequence; mitochondrial
163	OL588897.1	*Lutrogale perspicillata* isolate Sg_Besut_LP_1 D‐loop, partial sequence; mitochondrial
164	OL588896.1	*Lutrogale perspicillata* isolate Sg_Besut_LP_3 D‐loop, partial sequence; mitochondrial
165	OL588895.1	*Lutrogale perspicillata* isolate Sg_Besut_LP_2 D‐loop, partial sequence; mitochondrial
166	OL588894.1	*Lutrogale perspicillata* isolate Penarik_LP_7 D‐loop, partial sequence; mitochondrial
167	OL588893.1	*Lutrogale perspicillata* isolate Penarik_LP_10 D‐loop, partial sequence; mitochondrial
168	OL588892.1	*Lutrogale perspicillata* isolate Penarik_LP_8 D‐loop, partial sequence; mitochondrial
169	OL588891.1	*Lutrogale perspicillata* isolate Penarik_LP_9 D‐loop, partial sequence; mitochondrial
170	OL588890.1	*Lutrogale perspicillata* isolate Penarik_LP_12 D‐loop, partial sequence; mitochondrial
171	OL588889.1	*Lutrogale perspicillata* isolate Penarik_LP_11 D‐loop, partial sequence; mitochondrial
172	OL588888.1	*Lutrogale perspicillata* isolate Penarik_LP_6 D‐loop, partial sequence; mitochondrial
173	OL588887.1	*Lutrogale perspicillata* isolate Taman_Negara7_LP_6 D‐loop, partial sequence; mitochondrial
174	OL588886.1	*Lutrogale perspicillata* isolate Taman_Negara9_LP_3 D‐loop, partial sequence; mitochondrial
175	OL588885.1	*Lutrogale perspicillata* isolate Sg_Mersing_LP_2 D‐loop, partial sequence; mitochondrial
176	OL588884.1	*Lutrogale perspicillata* isolate Taman_Negara8_LP_2 D‐loop, partial sequence; mitochondrial
177	OL588883.1	*Lutrogale perspicillata* isolate Taman_Negara6_LP_3 D‐loop, partial sequence; mitochondrial
178	OL588882.1	*Lutrogale perspicillata* isolate Sg_Mersing_LP_4 D‐loop, partial sequence; mitochondrial
179	OL588881.1	*Lutrogale perspicillata* isolate Taman_Negara8_LP_1 D‐loop, partial sequence; mitochondrial
180	OL588880.1	*Lutrogale perspicillata* isolate Sg_Mersing_LP_6 D‐loop, partial sequence; mitochondrial
181	OL588879.1	*Lutrogale perspicillata* isolate Sg_Mersing_LP_5 D‐loop, partial sequence; mitochondrial
182	OL588878.1	*Lutrogale perspicillata* isolate Taman_Negara1_LP_1 D‐loop, partial sequence; mitochondrial
183	OL588877.1	*Lutrogale perspicillata* isolate Sg_Mersing_LP_1 D‐loop, partial sequence; mitochondrial
184	OL588876.1	*Lutrogale perspicillata* isolate Sg_Mersing_LP_3 D‐loop, partial sequence; mitochondrial
185	OL588875.1	*Lutrogale perspicillata* isolate Tg_Piai_LP_8 D‐loop, partial sequence; mitochondrial
186	OL588874.1	*Lutrogale perspicillata* isolate Tg_Piai_LP_5 D‐loop, partial sequence; mitochondrial
187	OL588873.1	*Lutrogale perspicillata* isolate Tg_Piai_LP_7 D‐loop, partial sequence; mitochondrial
188	OL588872.1	*Lutrogale perspicillata* isolate Tg_Piai_LP_6 D‐loop, partial sequence; mitochondrial
189	OL588871.1	*Lutrogale perspicillata* isolate Taman_Negara7_LP_4 D‐loop, partial sequence; mitochondrial
190	OL588870.1	*Lutrogale perspicillata* isolate Taman_Negara2_LP_1 D‐loop, partial sequence; mitochondrial
191	OL588869.1	*Lutrogale perspicillata* isolate Taman_Negara7_LP_5 D‐loop, partial sequence; mitochondrial
192	OL588868.1	*Lutrogale perspicillata* isolate Taman_Negara5_LP_2 D‐loop, partial sequence; mitochondrial
193	OL588867.1	*Lutrogale perspicillata* isolate Taman_Negara4_LP_1 D‐loop, partial sequence; mitochondrial
194	OL588866.1	*Lutrogale perspicillata* isolate Taman_Negara2_LP_2 D‐loop, partial sequence; mitochondrial
195	OL588865.1	*Lutrogale perspicillata* isolate Balok_LP_1 D‐loop, partial sequence; mitochondrial
196	OL588864.1	*Lutrogale perspicillata* isolate Temerloh_LP_3 D‐loop, partial sequence; mitochondrial
197	OL588863.1	*Lutrogale perspicillata* isolate Temerloh_LP_1 D‐loop, partial sequence; mitochondrial
198	OL588862.1	*Lutrogale perspicillata* isolate Temerloh_LP_2 D‐loop, partial sequence; mitochondrial
199	OL588861.1	*Lutrogale perspicillata* isolate Tg_Piandang8_LP_2 D‐loop, partial sequence; mitochondrial
200	OL588860.1	*Lutrogale perspicillata* isolate Tg_Lumpur_LP_1 D‐loop, partial sequence; mitochondrial
201	OL588859.1	*Lutrogale perspicillata* isolate Tg_Piandang5_LP_9 D‐loop, partial sequence; mitochondrial
202	OL588858.1	*Lutrogale perspicillata* isolate Tg_Piandang9_LP_5 D‐loop, partial sequence; mitochondrial
203	OL588857.1	*Lutrogale perspicillata* isolate Tg_Piandang4_LP_7 D‐loop, partial sequence; mitochondrial
204	OL588856.1	*Lutrogale perspicillata* isolate Kampar2_LP_3 D‐loop, partial sequence; mitochondrial
205	OL588855.1	*Lutrogale perspicillata* isolate Kampar2_LP_9 D‐loop, partial sequence; mitochondrial
206	OL588854.1	*Lutrogale perspicillata* isolate Kampar2_LP_5 D‐loop, partial sequence; mitochondrial
207	OL588853.1	*Lutrogale perspicillata* isolate Kuala_Gula2_LP_4 D‐loop, partial sequence; mitochondrial
208	OL588852.1	*Lutrogale perspicillata* isolate Kampar2_LP_4 D‐loop, partial sequence; mitochondrial
209	OL588851.1	*Lutrogale perspicillata* isolate Kampar2_LP_2 D‐loop, partial sequence; mitochondrial
210	OL588850.1	*Lutrogale perspicillata* isolate Kampar2_LP_12 D‐loop, partial sequence; mitochondrial
211	OL588849.1	*Lutrogale perspicillata* isolate Kampar2_LP_11 D‐loop, partial sequence; mitochondrial
212	OL588848.1	*Lutrogale perspicillata* isolate Kuala_Gula2_LP_9 D‐loop, partial sequence; mitochondrial
213	OL588847.1	*Lutrogale perspicillata* isolate Kampar2_LP_10 D‐loop, partial sequence; mitochondrial
214	OL588846.1	*Lutrogale perspicillata* isolate Kampar2_LP_8 D‐loop, partial sequence; mitochondrial
215	OL588845.1	*Lutrogale perspicillata* isolate Kampar2_LP_6 D‐loop, partial sequence; mitochondrial
216	OL588844.1	*Lutrogale perspicillata* isolate Kampar2_LP_7 D‐loop, partial sequence; mitochondrial
217	OL588843.1	*Lutrogale perspicillata* isolate Kampar2_LP_1 D‐loop, partial sequence; mitochondrial
218	OL588842.1	*Lutrogale perspicillata* isolate Kampar1_LP_7 D‐loop, partial sequence; mitochondrial
219	OL588841.1	*Lutrogale perspicillata* isolate Kampar1_LP_6 D‐loop, partial sequence; mitochondrial
220	OL588840.1	*Lutrogale perspicillata* isolate Kampar1_LP_5 D‐loop, partial sequence; mitochondrial
221	OL588839.1	*Lutrogale perspicillata* isolate Kampar1_LP_2 D‐loop, partial sequence; mitochondrial
222	OL588838.1	*Lutrogale perspicillata* isolate Kampar1_LP_3 D‐loop, partial sequence; mitochondrial
223	OL588837.1	*Lutrogale perspicillata* isolate Kuala_Gula1_LP_16 D‐loop, partial sequence; mitochondrial
224	OL588836.1	*Lutrogale perspicillata* isolate Kuala_Gula1_LP_19 D‐loop, partial sequence; mitochondrial
225	OL588835.1	*Lutrogale perspicillata* isolate Kampar1_LP_4 D‐loop, partial sequence; mitochondrial
226	OL588834.1	*Lutrogale perspicillata* isolate Tg_Piandang8_LP_3 D‐loop, partial sequence; mitochondrial
227	OL588833.1	*Lutrogale perspicillata* isolate Kampar1_LP_1 D‐loop, partial sequence; mitochondrial
228	OL588832.1	*Lutrogale perspicillata* isolate Kuala_Gula2_LP_2 D‐loop, partial sequence; mitochondrial
229	OL588831.1	*Lutrogale perspicillata* isolate Tanjung_Piandang3_LP_5 D‐loop, partial sequence; mitochondrial
230	OL588830.1	*Lutrogale perspicillata* isolate Tg_Piandang8_LP_1 D‐loop, partial sequence; mitochondrial
231	OL588829.1	*Lutrogale perspicillata* isolate Tg_Piandang9_LP_6 D‐loop, partial sequence; mitochondrial
232	OL588828.1	*Lutrogale perspicillata* isolate Kuala_Gula1_LP_15 D‐loop, partial sequence; mitochondrial
233	OL588827.1	*Lutrogale perspicillata* isolate Tg_Piandang9_LP_7 D‐loop, partial sequence; mitochondrial
234	OL588826.1	*Lutrogale perspicillata* isolate Tg_Piandang7_LP_14 D‐loop, partial sequence; mitochondrial
235	OL588825.1	*Lutrogale perspicillata* isolate Tg_Piandang9_LP_4 D‐loop, partial sequence; mitochondrial
236	OL588824.1	*Lutrogale perspicillata* isolate Tg_Piandang6_LP_13 D‐loop, partial sequence; mitochondrial
237	OL588823.1	*Lutrogale perspicillata* isolate Tanjung_Piandang3_LP_1 D‐loop, partial sequence; mitochondrial
238	OL588822.1	*Lutrogale perspicillata* isolate Tg_Piandang6_LP_11 D‐loop, partial sequence; mitochondrial
239	OL588821.1	*Lutrogale perspicillata* isolate T_Alam_LP_2 D‐loop, partial sequence; mitochondrial
240	OL588820.1	*Lutrogale perspicillata* isolate JUGRA_LP_3 D‐loop, partial sequence; mitochondrial
241	OL588819.1	*Lutrogale perspicillata* isolate Tg_Piandang5_LP_8 D‐loop, partial sequence; mitochondrial
242	OL588818.1	*Lutrogale perspicillata* isolate Panchang_Bedana1_LP_6 D‐loop, partial sequence; mitochondrial
243	OL588817.1	*Lutrogale perspicillata* isolate Tanjung_Piandang3_LP_6 D‐loop, partial sequence; mitochondrial
244	OL588816.1	*Lutrogale perspicillata* isolate Panchang_Bedana3_LP_21 D‐loop, partial sequence; mitochondrial
245	OL588815.1	*Lutrogale perspicillata* isolate Tanjung_Piandang3_LP_4 D‐loop, partial sequence; mitochondrial
246	OL588814.1	*Lutrogale perspicillata* isolate Tanjung_Piandang3_LP_2 D‐loop, partial sequence; mitochondrial
247	OL588813.1	*Lutrogale perspicillata* isolate Tanjung_Piandang3_LP_3 D‐loop, partial sequence; mitochondrial
248	OL588812.1	*Lutrogale perspicillata* isolate Tg_Piandang2_LP_5 D‐loop, partial sequence; mitochondrial
249	OL588811.1	*Lutrogale perspicillata* isolate T_Alam_LP_3 D‐loop, partial sequence; mitochondrial
250	OL588810.1	*Lutrogale perspicillata* isolate Kelanang_LP‐2 D‐loop, partial sequence; mitochondrial
251	OL588809.1	*Lutrogale perspicillata* isolate Tg_Piandang2_LP_8 D‐loop, partial sequence; mitochondrial
252	OL588808.1	*Lutrogale perspicillata* isolate Tg_Piandang2_LP_11 D‐loop, partial sequence; mitochondrial
253	OL588807.1	*Lutrogale perspicillata* isolate Paya_Indah2_LP_8 D‐loop, partial sequence; mitochondrial
254	OL588806.1	*Lutrogale perspicillata* isolate Panchang_Bedana3_LP_9 D‐loop, partial sequence; mitochondrial
255	OL588805.1	*Lutrogale perspicillata* isolate Paya_Indah2_LP_9 D‐loop, partial sequence; mitochondrial
256	OL588804.1	*Lutrogale perspicillata* isolate Sg_Dinding_LP_2 D‐loop, partial sequence; mitochondrial
257	OL588803.1	*Lutrogale perspicillata* isolate M_Nawar2_LP_5 D‐loop, partial sequence; mitochondrial
258	OL588802.1	*Lutrogale perspicillata* isolate Pergau_2_LP_3 D‐loop, partial sequence; mitochondrial
259	OL588801.1	*Lutrogale perspicillata* isolate P_Lalang_LP_3 D‐loop, partial sequence; mitochondrial
260	OL588800.1	*Lutrogale perspicillata* isolate P_Lalang_LP_1 D‐loop, partial sequence; mitochondrial
261	OL588799.1	*Lutrogale perspicillata* isolate Cenderoh_2_LP_2 D‐loop, partial sequence; mitochondrial
262	OL588798.1	*Lutrogale perspicillata* isolate P_Lalang_LP_2 D‐loop, partial sequence; mitochondrial
263	OL588797.1	*Lutrogale perspicillata* isolate Tumpat_LP_1 D‐loop, partial sequence; mitochondrial
264	OL588796.1	*Lutrogale perspicillata* isolate K_Salang_LP_2 D‐loop, partial sequence; mitochondrial
265	OL588795.1	*Lutrogale perspicillata* isolate M_Nawar1_LP_2 D‐loop, partial sequence; mitochondrial
266	OL588794.1	*Lutrogale perspicillata* isolate Kuala_Besar_LP_3 D‐loop, partial sequence; mitochondrial
267	OL588793.1	*Lutrogale perspicillata* isolate Kuala_Besar_LP_1 D‐loop, partial sequence; mitochondrial
268	OL588792.1	*Lutrogale perspicillata* isolate Sg_Dinding_LP_3 D‐loop, partial sequence; mitochondrial
269	OL588791.1	*Lutrogale perspicillata* isolate Tg_Batu_LP_5 D‐loop, partial sequence; mitochondrial
270	OL588790.1	*Lutrogale perspicillata* isolate Tg_Batu_LP_4 D‐loop, partial sequence; mitochondrial
271	OL588789.1	*Lutrogale perspicillata* isolate Tg_Batu_LP_3 D‐loop, partial sequence; mitochondrial
272	OL588788.1	*Lutrogale perspicillata* isolate T_MUDA_LP_3 D‐loop, partial sequence; mitochondrial
273	OL588787.1	*Lutrogale perspicillata* isolate T_MUDA_LP_2 D‐loop, partial sequence; mitochondrial
274	OL588786.1	*Lutrogale perspicillata* isolate BELUM1_LP_2 D‐loop, partial sequence; mitochondrial
275	OL588785.1	*Lutrogale perspicillata* isolate BELUM2_LP_1 D‐loop, partial sequence; mitochondrial
276	OL588784.1	*Lutrogale perspicillata* isolate Merlimau_LP_2 D‐loop, partial sequence; mitochondrial
277	OL588783.1	*Lutrogale perspicillata* isolate YAN_LP_1 D‐loop, partial sequence; mitochondrial
278	OL588782.1	*Lutrogale perspicillata* isolate Merlimau_LP_1 D‐loop, partial sequence; mitochondrial
279	OL588781.1	*Lutrogale perspicillata* isolate M_Nawar2_LP_8 D‐loop, partial sequence; mitochondrial
280	OL588780.1	*Lutrogale perspicillata* isolate ALAI_LP_4 D‐loop, partial sequence; mitochondrial
281	OL588779.1	*Lutrogale perspicillata* isolate T_MUDA_LP_4 D‐loop, partial sequence; mitochondrial
282	OL588778.1	*Lutrogale perspicillata* isolate BELUM1_LP_1 D‐loop, partial sequence; mitochondrial
283	OL588777.1	*Lutrogale perspicillata* isolate Tlk_Kampi_LP_3 D‐loop, partial sequence; mitochondrial
284	OL588776.1	*Lutrogale perspicillata* isolate BELUM2_LP_2 D‐loop, partial sequence; mitochondrial
285	OL588775.1	*Lutrogale perspicillata* isolate Tlk_Kampi_LP_2 D‐loop, partial sequence; mitochondrial
286	OL588774.1	*Lutrogale perspicillata* isolate Cenderoh_3_LP_3 D‐loop, partial sequence; mitochondrial
287	OL588773.1	*Lutrogale perspicillata* isolate Bayram_LP_1 D‐loop, partial sequence; mitochondrial
288	OL588772.1	*Lutrogale perspicillata* isolate Bayram_LP_2 D‐loop, partial sequence; mitochondrial
289	OL588771.1	*Lutrogale perspicillata* isolate ALAI_LP_3 D‐loop, partial sequence; mitochondrial
290	OL588770.1	*Lutrogale perspicillata* isolate ALAI_LP_1 D‐loop, partial sequence; mitochondrial
291	OL588769.1	*Lutrogale perspicillata* isolate Sg_Dinding_LP_4 D‐loop, partial sequence; mitochondrial
292	OL588768.1	*Lutrogale perspicillata* isolate M_Nawar2_LP_6 D‐loop, partial sequence; mitochondrial
293	OL588767.1	*Lutrogale perspicillata* isolate TB_MERAH_LP_4 D‐loop, partial sequence; mitochondrial
294	OL588766.1	*Lutrogale perspicillata* isolate Cenderoh_2_LP_3 D‐loop, partial sequence; mitochondrial
295	OL588765.1	*Lutrogale perspicillata* isolate BIKAM_LP_1 D‐loop, partial sequence; mitochondrial
296	OL588764.1	*Lutrogale perspicillata* isolate Klinggi_NS_LP_2 D‐loop, partial sequence; mitochondrial
297	OL588763.1	*Lutrogale perspicillata* isolate Cenderoh_2_LP_1 D‐loop, partial sequence; mitochondrial
298	OL588762.1	*Lutrogale perspicillata* isolate M_Nawar2_LP_7 D‐loop, partial sequence; mitochondrial
299	OL588761.1	*Lutrogale perspicillata* isolate Kuala_Gula2_LP_5 D‐loop, partial sequence; mitochondrial
300	OL588760.1	*Lutrogale perspicillata* isolate Pergau_2_LP_2 D‐loop, partial sequence; mitochondrial
301	OL588759.1	*Lutrogale perspicillata* isolate BELUM2_LP_3 D‐loop, partial sequence; mitochondrial
302	OL588758.1	*Lutrogale perspicillata* isolate YAN_LP_2 D‐loop, partial sequence; mitochondrial
303	OL588757.1	*Lutrogale perspicillata* isolate Tg_Batu_LP_6 D‐loop, partial sequence; mitochondrial
304	OL588756.1	*Lutrogale perspicillata* isolate K_Salang_LP_1 D‐loop, partial sequence; mitochondrial
305	OL588755.1	*Lutrogale perspicillata* isolate Klinggi_NS_LP_1 D‐loop, partial sequence; mitochondrial
306	OL588754.1	*Lutrogale perspicillata* isolate Tg_Piandang5_LP_10 D‐loop, partial sequence; mitochondrial
307	OL588753.1	*Lutrogale perspicillata* isolate ALAI_LP_2 D‐loop, partial sequence; mitochondrial
308	OL588752.1	*Lutrogale perspicillata* isolate Temengor2_LP_1 D‐loop, partial sequence; mitochondrial
309	OL588751.1	*Lutrogale perspicillata* isolate M_Nawar1_LP_1 D‐loop, partial sequence; mitochondrial
310	OL588750.1	*Lutrogale perspicillata* isolate Bkt_Malut_LP_1 D‐loop, partial sequence; mitochondrial
311	OL588749.1	*Lutrogale perspicillata* isolate Pokok_Sena_LP_2 D‐loop, partial sequence; mitochondrial
312	OL588748.1	*Lutrogale perspicillata* isolate Bkt_Malut_LP_2 D‐loop, partial sequence; mitochondrial
313	OL588747.1	*Lutrogale perspicillata* isolate Pokok_Sena_LP_3 D‐loop, partial sequence; mitochondrial
314	OL588746.1	*Lutrogale perspicillata* isolate Pokok_Sena_LP_1 D‐loop, partial sequence; mitochondrial
315	OL588745.1	*Lutrogale perspicillata* isolate Temengor2_LP_2 D‐loop, partial sequence; mitochondrial
316	OL588744.1	*Lutrogale perspicillata* isolate Temengor5_LP_1 D‐loop, partial sequence; mitochondrial
317	OL588638.1	*Lutra sumatrana* isolate Tumpat_LS D‐loop, partial sequence; mitochondrial
318	OL588637.1	*Lutra sumatrana* isolate Tasik_Bera_LS D‐loop, partial sequence; mitochondrial
319	OL588636.1	*Lutra sumatrana* isolate Pontian_LS D‐loop, partial sequence; mitochondrial
320	OL588635.1	*Lutra sumatrana* isolate Pekan_LS D‐loop, partial sequence; mitochondrial
321	OL588634.1	*Lutra sumatrana* isolate Paya_Indah_LS D‐loop, partial sequence; mitochondrial
322	OL588633.1	*Lutra sumatrana* isolate Muazzam_LS D‐loop, partial sequence; mitochondrial
323	OL588632.1	*Lutra sumatrana* isolate Gopeng_LS D‐loop, partial sequence; mitochondrial
324	OL588632.1	*Lutra sumatrana* isolate Gopeng_LS D‐loop, partial sequence; mitochondrial

		Complete mitochondrial genome
S.No.	GenBank accession number	Description
1	KY117556.1	*Lutra sumatrana* isolate 49 mitochondrion, complete genome
2	KY117557.1	*Lutrogale perspicillata* isolate 21mitochondrion, complete genome
3	KY117535.1	*Aonyx cinerea* isolate 16 mitochondrion, complete genome
