# Supplementary material for: Identification of three Asian otter species (Aonyx cinereus, Lutra sumatrana, and Lutrogale perspicillata) using a novel noninvasive PCR‐RFLP analysis
Source: Ecol Evol. 2022 Dec 12;12(12):e9585. doi: 10.1002/ece3.9585 (PMC9743061; doi:10.1002/ece3.9585)
Supplement: Supplementary file 1 — Table S1. [file ECE3-12-e9585-s001.docx]

Suppl Table 1: Information about reference samples of various species used in this study.
